# Cell-Penetrating Peptide Derived from Human Eosinophil Cationic Protein Inhibits Mite Allergen Der p 2 Induced Inflammasome Activation

**DOI:** 10.1371/journal.pone.0121393

**Published:** 2015-03-25

**Authors:** Sheng-Jie Yu, En-Chih Liao, Meei-Ling Sheu, Dah-Tsyr Margaret Chang, Jaw-Ji Tsai

**Affiliations:** 1 Institute of Biomedical Sciences, National Chung Hsing University, Taichung, Taiwan; 2 Department of Medical Research, Taichung Veterans General Hospital, Taichung, Taiwan; 3 Department of BioIndustry Technology, Da Yeh University, Changhua, Taiwan; 4 Department of Medical Technology, Jen Ten College of Medicine, Nursing and Management, Miaoli, Taiwan; 5 Institute of Molecular and Cellular Biology, National Tsing Hua University, Hsinchu, Taiwan; 6 Section of Allergy, Immunology and Rheumatology, Department of Internal Medicine, Taichung Veterans General Hospital, Taichung, Taiwan; 7 Institute of Clinical Medicine, National Yang Ming University, Taipei, Taiwan; French National Centre for Scientific Research, FRANCE

## Abstract

Newly discovered cell penetration peptides derived from human eosinophil cationic proteins (CPPecp) have the characteristic of cell internalization, but the effect of CPPecp on immunomodulation has not been clarified. House dust mite (HDM) major allergen, Der p 2, can induce proinflammatory cytokine production which contributes to airway inflammation and allergic asthma. However, the mechanism of Der p 2 on NLRP3 inflammasome activation remains unclear. The aim of this study was to investigate the immunomodulatory effect of CPPecp on inhibition of Der p 2 induced inflammasome activation. We showed that proinflammatory cytokines IL-1β, IL-6 and IL-8 were significantly upregulated in peripheral blood mononuclear cells (PBMCs) derived from HDM allergic patients after Der p 2 stimulation. Expression of NLRP3, ASC, Caspase-1, IL-1β and Caspase-1 activity was upregulated in THP-1 cells after Der p 2 stimulation. Proinflammatory cytokine production, NLRP3 inflammasome activation and caspase-1 activity were downregulated in THP-1 cells and CD14+ cells co-cultured with Der p 2 and CPPecp. The immunomodulatory effect of CPPecp was through upregulation of IFN-α production but not induction of autophagy. These results suggested Der p 2 plays an important role in NLRP3 inflammasome activation and CPPecp has the potential to be a novel anti-inflammatory agent for allergic inflammation treatment in the future.

## Introduction

House dust mite (HDM) allergy has been strongly associated with chronic airway inflammation and allergic asthma [1, 2]. More than 50% of children and adolescents with asthma are sensitized to HDM [3]. The most common species of HDM are *Dermatophagoides pteronyssinus*. Although some major groups of HDM allergens have been identified, the most notable is Der p 2 [4]. Fecal particles contain the most allergens and the highest dust mite concentrations are found in mattresses. It has been shown that dust mite exposure in early childhood is an important determinant in asthma development [5]. Allergic inflammation results not only from an exacerbated Th2-biased adaptive immune response but is heavily influenced by the direct activation of the innate immune cells such as bronchial epithelial cells, dendritic cells, mast cells, monocytes and eosinophils by both the allergens themselves and danger signals present in the allergen sources [6].

The importance of HDM in innate immunity was first reported in 2009[7]. The innate immune response detects pathogen or allergen invasive signals whether foreign or intrinsic. Pattern-recognition receptors (PRRs) mediate the detection of bacterial cell components including toll-like receptors (TLRs), RIG-I-like receptors (RLRs), and nucleotide-binding domain and leucine-rich repeat proteins (NLRs) [8]. Pathogens activate the transcriptional and translational induction of a range of proinflammatory cytokines, but also elicit the activation of a multimeric protein complex known as the inflammasome that is critical in the proteolytic processing of pro- IL-1β and pro-IL-18 into their mature active forms [9, 10]. The inflammasome is classically composed of an NLR, the adaptor molecule PYCARD/ASC, and pro-caspase-1, which when proteolyzed to caspase-1 provides the enzymatic activity of the inflammasome [11].

Immunotherapy is defined as the “treatment of disease by inducing, enhancing, or suppressing an immune response” [12, 13]. Immunotherapies designed to elicit or amplify an immune response are classified as activation immunotherapies. Cell penetrating peptide (CPP) has been used to treat allergic diseases in recent years[14, 15]. The first CPP to be identified was Tat peptide in 1989, corresponding to the basic domain of HIV-1 Tat protein[16]. Penetratin, corresponding to the third helix of the Antennapedia homeodomain, was identified in 1994 [17]. Since then, various peptides showing the same capacities have been identified or rationally designed. In this study, 10-residue peptide-CPPecp was derived from the human eosinophil cationic protein (ECP). ECP is a secretory ribonuclease (RNase) released by activated eosinophils and it has antiviral and antiparasitic activities [18]. In addition, ECP binds lipopolysaccharides and peptidoglycans tightly [19]. The N-terminal domain of ECP (residues 1–45) retains most of the antimicrobial properties [20]. CPPecp has been shown to internalize into bronchial epithelial cells [21]. However, to our knowledge, the effects of CPPecp on immunomodulation have not been reported. The aims of this study were to investigate the mechanism of Der p 2 involvement in inflammasome activation and the inhibitory effects of CPPecp on immunomodulation of Der p 2-induced inflammasome activation.

## Materials and Methods

### Cells culture

THP-1 cells were a kind gift from Dr. Meei Ling Sheu (National Chung Hsing University, Taiwan).THP-1 cells were cultured in RPMI 1640 medium, 10% (v/v) heat-inactivated fetal bovine serum (FBS), and 1% streptomycin/penicillin (Thermo, New York, USA). Cellular differentiation of suspended monocytes to adherent macrophages was induced by overnight culture in complete medium supplemented with 300ng/ml phorbol 12-myristate13-acetate (PMA), followed by culture in complete medium for one day. In PBMCs culture, 16-mL blood samples were collected and the PBMCs were separated by density centrifugation using the Ficoll-Paque Plus density gradient (Pharmacia Biotech, Freiburg, Germany) [22]. Cells were maintained in RPMI-1640 medium containing 10% heat inactivated FBS and 1% streptomycin/penicillin in a humidified 5% CO2 atmosphere. The study was approved by the Research Ethics Committee of Taichung Veterans General Hospital.

### Purification of CD14^+^ cells

CD14^+^ cells were purified from PBMCs by negative selection with a monocyte isolation kit (STEMCELL, New York, USA) containing bispecific tetrameric antibody complexes which are directed against cell surface antigens on human blood cells (CD2, CD3, CD19, CD20, CD56, CD66b, CD123, glycophorin A and dextran), according to the manufacturer’s instructions.

### Selection of patients

Base on GINA guidelines 2014, asthma is characterized by chronic airway inflammation and defined by history of respiratory syndromes such as wheeze, shortness of breath, chest tightness and cough that vary over time and in intensity, together with variable expiratory airflow limitation. In this study, patients recruited in this study were defined as a history of reversible obstructive airway disease associated with exacerbation resulting from allergen exposure and concomitant skin reactivity to exacerbating allergens. All patients were sensitive to mites and had mite-specific IgE as determined using the Phadia CAP system (Thermo Fisher Scientific, Uppsala, Sweden). All subjects were selected from the clinic of the Division of Allergy, Immunology and rheumatology of Taichung Veterans General Hospital.

### Ethics statement

All subjects provided written informed consent, and the protocols and all research involving human participants were approved by the Institutional Review Board of Taichung Veterans General Hospital (TCVGH-CF12010#1).

### Peptide synthesis

CPPecp (NYRWRCKNQN, 1381Da) was synthesized at Angene Biotech Co., Ltd., Taiwan, and the purity (>90%) was assessed by analytical high-performance liquid chromatography. Peptide sequences were confirmed by matrix-assisted laser desorption/ionisation time-of-flight mass spectrometry at Angene Biotech Co., Ltd., Taiwan.

### Der p 2 preparation

Purified recombinant protein Der p 2 (RP-DP2C-1) was purchased from Indoor Biotechnologies (Charlottesville, Virginia, USA).

### Cell viability was determined by trypan blue dye exclusion

THP-1 cells were treated with 10 and 100uM CPPecp for three days. After the treatment, the cells were collected and resuspended in the culture medium. After cells were mixed 1:1 with trypan blue (Biological Industries, Kibbutz Beit Haemek, Israel), they were counted using a hemocytometer. The cell viability is calculated as the number of viable cells divided by the total number of cells.

### Staining with autophagosomal small molecule probes

Autophagosomes were detected by a Cyto-ID staining kit (ENZO Life Science, New York, USA) according to the manufacturer’s protocol [23]. THP-1 cells grown on 12-well plates, cells were treated with 100uM of CPPecp at 37°C for a time course study, and the cells were also treated with 10uM of tamoxifen for six hours was used as autophagy induction positive control. After treatment, the medium was removed with testing reagents and positive control and the cells were washed twice with 1X assay buffer. One hundred μL of Microscopy Dual Detection Reagent was dispensed to cover each sample of monolayer cells. Samples were kept in the dark and incubated for 15–30 minutes at 37°C. Cells were then washed with 100 μL of 1X assay buffer. Stained cells were analyzed by flow cytometry (BD FACSCalibur).

### Caspase-1 activity

The caspase-1 activity was detected using a Caspase-1 colorimetric assay kit (R&D Systems, Minnesota, USA), according to the manufacturer’s protocol. Briefly, after stimulation, THP-1 cells were collected by centrifugation at 250 g at 4°C for 10 min. The cell pellet was lysed by the addition 50ul of the lysis buffer. The cell lysate was incubate on ice for 10 min, and then centrifuged at 10,000g for 1 min. Supernatants were then mixed with 50 ul of 2x assay buffer in 96 well flat bottom plate followed by addition of 5 ul caspase-1 colorimetric substrate (WEHD pNA), and incubation at 37°C for two hours. The optical density was measured at 405 nm with a TECAN Sunrise ELISA reader.

### Enzyme-linked immunosorbent assay (ELISA)

THP-1 cells, PBMCs and CD14^+^ cells were treated with 1.5ug/ml of Der p 2 or co-cultured with different dose (10, 50, 100 uM) CPPecp for different incubation periods. After treatment, the supernatants were collected, centrifuged, and analyzed for IL-1β, IL-6, IL-8 and IFN-β production using commercial enzyme-linked immunosorbent assay kits (R&D Systems, Minnesota, USA; BioLegend, San Diego, USA; PBL assay science, New Jersey, USA) according to the manufacturer's instructions.

### Reverse transcription-polymerase chain reaction (RT-PCR)

The total RNA was isolated using an RNeasy mini kit (Qiagen, Manchester, Germany) according to the manufacturer’s protocol. The first standard cDNA was synthesized by the extension of oligo(dT)^18^ primers with 40 units of M-MLV Reverse Transcriptase (Thermo, New York, USA) in a mixture containing 1 ug of total RNA. The cDNA served as a template in a PCR using a G-STORM Thermal Cycler. The primers used were: 5’- AAACAGTGAAGTGCTCCTTCCAGG-3’ and 5’- TGGAGAACACCACTTGTTGCTCCA-3’ for IL-1β; 5’- GAGAAAGGAGACATGTAACAAGAGT-3’ and 5’- GCGCAGAATGAGATGAGTTGT-3’ for IL-6; 5’- TTGGCAGCCTTCCTGATTTCT-3’ and 5’- TCTCAGCCCTCTTCAAAAACTTCTC-3’ for IL-8; 5’-CCTAGACAAATTCTGCACCG-3’ and 5’-TCATAGTTATAGCAGGGGTGAG-3’ for IFN-α; and 5’- CCACCCATGGCAAATTCCATGGCA and 5’-TCTAGACGGCAGGTCAGGTCCACC-3’ for GAPDH. GAPDH was used as an internal control. The PCR products were analyzed with 2% agarose gel electrophoresis and ethidium bromide staining. Images captured and analyzed was used Kodak molecular imaging system (Kodak, NY, USA).

### Western blotting

Whole cell lysates were prepared as described previously [24, 25]. 10ug total protein with 2x sample buffer was loaded per lane. After blocking, the blots were incubated with antibodies for anti-human NLRP3, ASC, caspase-1, (Cell Signaling, Massachusetts, USA; Santa Cruz Biotechnology, Texas, USA) andβ-actin (Millipore, Massachusetts, USA) in TBS with 0.1% Tween 20 overnight at 4°C followed by three 10-min washes in TBS with 0.1% Tween 20. The membranes were then incubated with horseradish peroxidase-conjugated secondary antibodies (Millipore, Massachusetts, USA) for one hour. Detection was performed with ECL (Millipore, Massachusetts, USA), and chemiluminescence was detected by LAS 3000. Band intensity was analyzed by Multi Gauge software V 3.0.

### Statistical analysis

Statistical analyses were performed using GraphPad Prism 5 (GraphPad Software, San Diego, CA, USA). Data are presented as mean ± standard error of the mean (SEM). P-values ≤0.05 were considered statistically significant. All the results from THP-1 cells were compared between different groups of treatment and analyzed by the Mann-Whitney test. All the experiences of PBMCs and CD14+ cells were compared between groups treatment and analyzed by pair student’s t test.

## Results

### Der p 2 upregulates pro-inflammatory cytokine expression in human PBMCs

**Fig 1:**
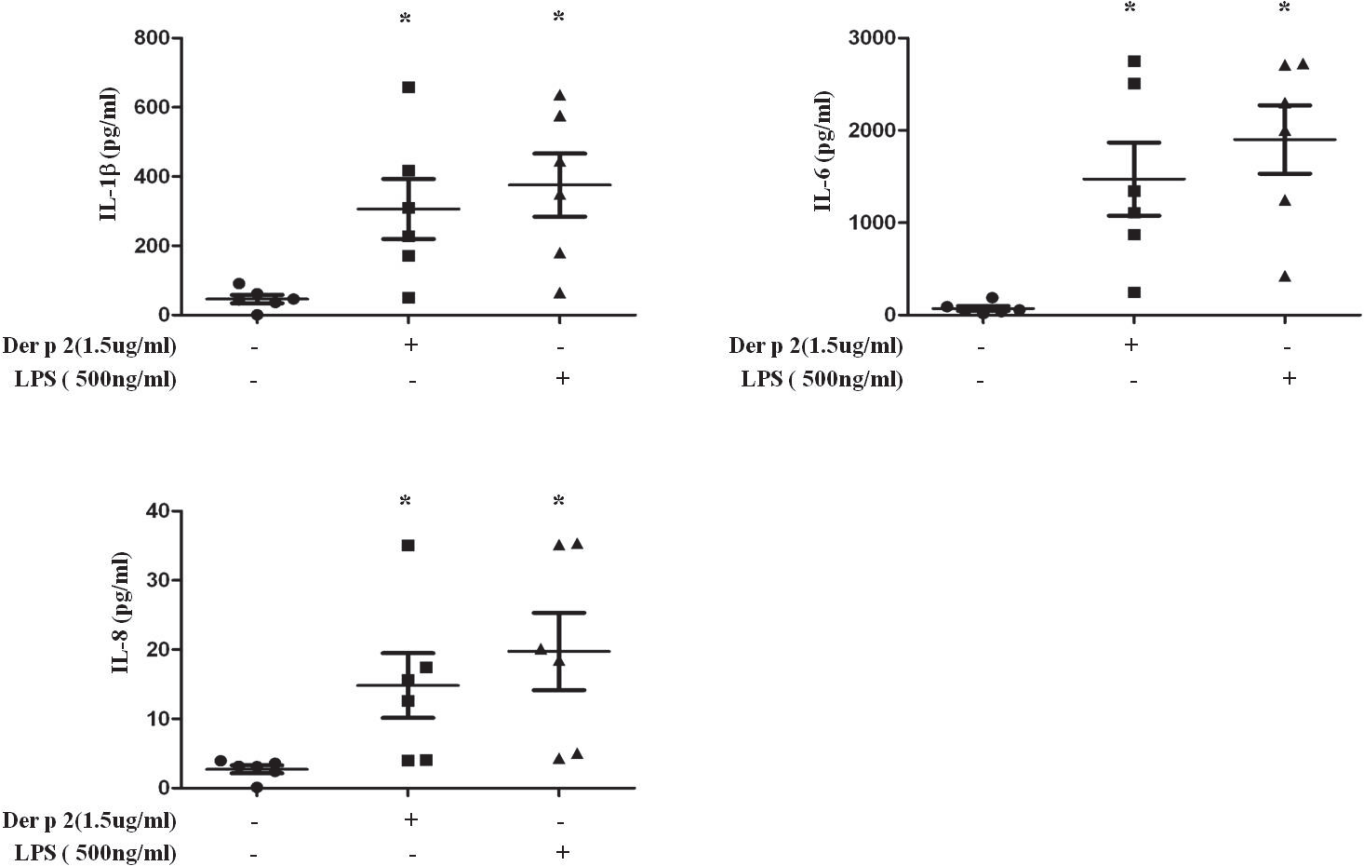
Der p 2 induces pro-inflammatory cytokine expression in HDM allergic patients. PBMCs derived from HDM allergic patients (n = 6) were stimulated with Der p 2 (1.5ug/ml) for six hours; LPS (500ng/ml) was used as control. After stimulation, the culture supernatant was collected and protein levels of IL-1β, IL-6 and IL-8 were measured by ELISA. Bars and error bars indicate mean and standard error of the mean (SEM), respectively. * p<0.05 compared to control group.

Allergen induced pro-inflammatory cytokine production is associated with allergic inflammation. We investigated the correlation between mite major allergen-Der p 2 and allergic inflammation by culturing PBMCs derived from six HDM allergic subjects cultured with Der p 2 (1.5ug/ml) or LPS (500ng/ml) for six hours. Culture supernatant was collected for cytokine measurement by ELISA. The results showed that IL-1β, IL-6 and IL-8 were significantly upregulated after Der p 2 stimulation (p<0.05; Fig. 1). Furthermore, we investigated the effect of Der p 2 is specific to HDM allergic patients, CD14^+^ cells derived from two non HDM allergic patients were cultured with Der p 2 (1.5ug/ml) or LPS (500ng/ml) for six hours. Secreted IL-1β, IL-6 and IL-8 in the culture supernatant were not increase after Der p 2 stumulation (S1 Fig.)

### Der p 2 induces IL-1β expression through inflammasome activation in monocytes

**Fig 2:**
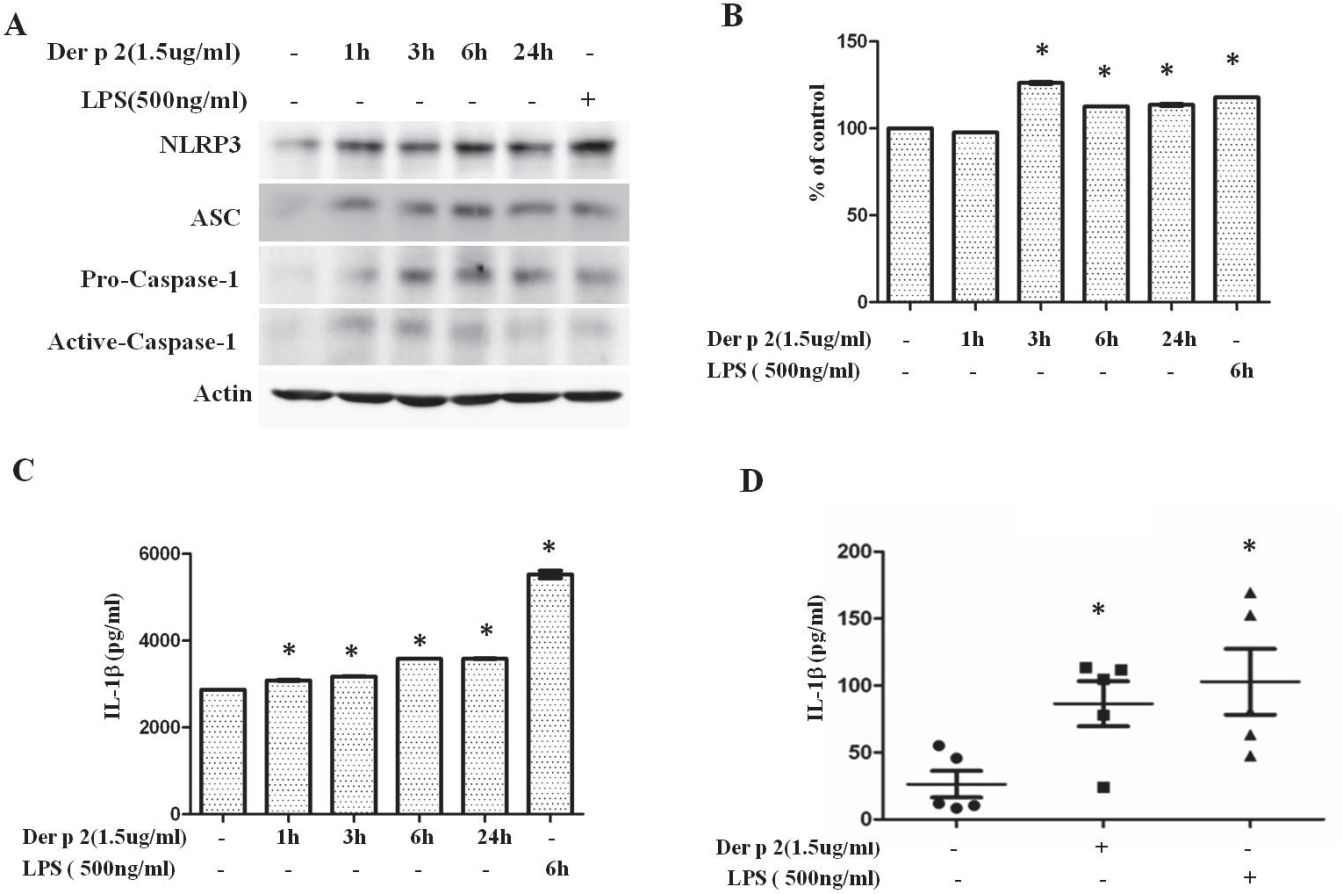
Inflammasome activation can be induced by Der p 2 stimulation. (A) THP-1 cells were treated with Der p 2 (1.5ug/ml) at different time points. Cells treated with LPS (500ng/ml) for six hours were used as positive control. After stimulation, cell lysates were collected and separated with SDS-PAGE. Expressions of NLRP3, ASC and caspase-1 were detected by Western blot. Caspase-1 enzymatic activity was assayed in cell lysates of THP-1 cells stimulated with Der p 2 (1.5ug/ml) at different time points. Caspase-1activities were expressed as percent of control with *p-value <0.05 compared to control (B). IL-1β production from (C) THP-1 cells and (D) CD14^+^ cells derived from HDM allergic subjects (n = 5) were measured by ELISA. Bars and error bars indicate mean and standard error of the mean (SEM), respectively. * p<0.05, compared to control. Results shown are representative of three independent experiments.

The proinflammatory cytokine IL-1β is involved in allergic airway inflammation. In addition, its production is correlated with inflammasome activation. Components of the NLRP3 inflammasome complex and pro-inflammatory cytokines were investigated for the purpose of analyzing whether IL-1β production was through activated NLRP3 inflammasome. Cells from a human monocyte cell line, THP-1 cells, were used to evaluate Der p 2-induced inflammasome activation and IL-1β production. THP-1 cells were cultured with Der p 2 (1.5ug/ml) from one to twenty-four hours to investigate the effects of Der p 2 on inflammasome activation. The results showed that expression levels of NLRP3, ASC and caspase-1β were upregulated after Der p 2 stimulation (Fig. 2A). Classically, inflammasome complex assembly, accompanied by caspase-1 activation resulted in secretion of IL-1β. Thus, we further investigated caspase-1 activity. The result showed that THP-1 cells after treated with Der p 2 (1.5 ug/ml) for three hours, caspase-1 activity was significantly upregulate (Fig. 2B). IL-1βELISAβ data confirmed NLRP3 inflammasome were activated in THP-1 cells after induced by Der p 2 stimulation. The results showed that Der p 2 could significantly upregulate IL-1β production in a time dependent manner (p<0.05; Fig. 2C). We also used primary human CD14^+^ cells to investigate the IL-1β production. These results showed that concentration of IL-1β was significantly upregulated after Der p 2 stimulation (p<0.05; Fig. 2D). These data indicate that Der p 2 can induce inflammasome activation and in turn increase production of proinflammatory cytokines.

### CPPecp downregulates Der p 2-induced inflammasome activation in monocytes and production of pro-inflammatory cytokines in PBMCs

**Fig 3:**
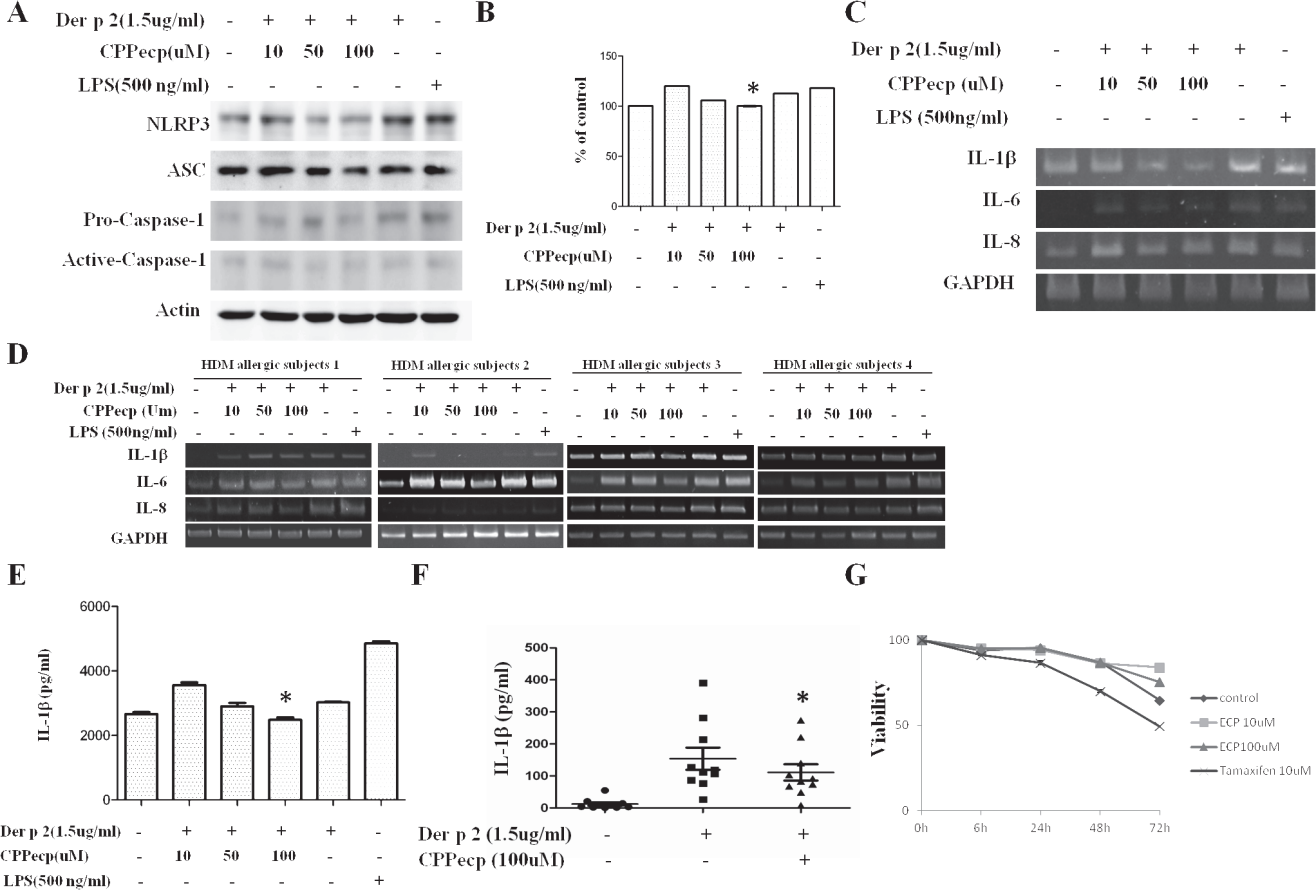
CPPecp inhibits inflammasome activation and downregulates proinflammatory cytokine production. (A) THP-1 cells were co-cultured with Der p 2 (1.5ug/ml) and CPPecp (10 to 100 uM) for 6 hours; LPS (500ng/ml) was used as control. Protein lysates were collected and expressions of NLRP3, ASC, caspase-1 was detected by Western blot. Caspase-1 enzymatic activity was assayed in cell lysates of THP-1 cells stimulated with Der p 2 (1.5ug/ml) co-cultured with CPPecp (10 to 100 uM) for 6 hours; LPS (500ng/ml) was used as control. Caspase-1activities were expressed as percent of control with *p-value <0.05 compared to treated with Der p 2 treatment group (B). THP-1 cells (C) and CD14^+^(D) cells co-cultured with Der p 2 (1.5ug/ml) and CPPecp (10 to 100 uM) for 6 hours, mRNA expression of IL-1β, IL-6, IL-8 and GAPDH were detected by RT-PCR. THP-1 cells (E) were co-cultured with CPPecp (10 to 100 uM) and PBMCs (F) derived from HDM allergic patients (n = 10) were co-cultured with Der p 2 1.5ug/ml and CPPecp 100uM for six hours. Culture supernatant was collected and IL-1β concentration was measured by ELISA. Bars and error bars indicate mean and standard error of the mean (SEM), respectively. * p<0.05 compared to treated with Der p 2 treatment group. THP-1 cells (G) were treated with 10 and 100uM CPPecp for different incubation periods. After treatment, cell viability was measured by trypan blue exclusion. Tamaxifen (10uM) was used as control. Results shown are representative of three independent experiments.

The inhibitory effect of CPPecp on Der p 2-induced inflammasome activation was investigated. THP-1 cells were co-cultured with CPPecp (10–100uM) and Der p 2 (1.5ug/ml) or LPS (500ng/ml) for six hours followed by analysis of inflammasome activation. The results showed that Der p 2 induced overexpression of NLRP3 and caspase-1 was significantly downregulated by CPPecp at a concentration of 100uM. However, expression of ASC was only slightly decreased (Fig. 3A). We also used CPPecp (10, 50, 100uM) alone to treat THP-1 cells for six hours as control to investigate the effect of CPPecp on inflammasome activation. The result showed that NLRP3, ASC and Caspase-1 were not upregulated after CPPecp treatment and secreted IL-1β and IL-6 in the culture supernatant were not increase (S2A and B Fig.). In addition, caspase-1 activity was also investigated and the result showed that caspase-1 activity was significantly decreased after cells co-cultured with Der p 2 (1.5ug/ml) and CPPecp (100uM) (p<0.05, Fig. 3B). Using RT-PCR, we further analyzed the effects of CPPecp on pro-inflammatory cytokine expression in THP-1 cells and CD14^+^ cells derived from HDM allergic subjects by RT-PCR. The results showed that expressions of IL-1β, IL-6 and IL-8 were downregulated after cells were co-cultured with Der p 2 (1.5ug/ml) and CPPecp at a concentration of 100uM (Fig. 3C and 3D respectively). CD14^+^ cells derived from two non HDM allergic subjects were also include in this study. CD14^+^ cells were co-cultured with Der p 2 (1.5ug/ml) and CPPecp with different concentration (10, 50, 100uM). The result showed that expression of IL-1β, IL-6 and IL-8 were not altered (S3 Fig.). In addition, we investigated the effect of CPPecp on Der p 2-induced IL-1β production fromTHP-1 cells and PBMCs derived from HDM allergic subjects. THP-1 cells were co-cultured with different concentration of CPPecp (10–100uM) and Der p 2 (1.5ug/ml) for six hours; PBMCs were co-cultured with Der p 2 (1.5ug/ml) and CPPecp (100uM) for six hours, then culture supernatant was collected and a concentration of IL-1β was measured by ELISA. The result showed that the concentration of IL-1β was significantly downregulated in THP-1 cells and PBMCs when Der p 2 co-cultured with 100uM of CPPecp (Fig. 3E and 3F respectively). The cytotoxic effect of CPPecp on THP-1 cells was also investigated. THP-1 cells were cultured with different concentrations of CPPecp (10uM and 100uM) for three days. The results showed that there was no cytotoxicity effect after forty-eight hours, and the viability was above 90% at both tested concentrations (Fig. 3G).

### CPPecp downregulates Der p 2-induced inflammasome activation through upregulation of IFN-β production but not induction of autophagy

**Fig 4:**
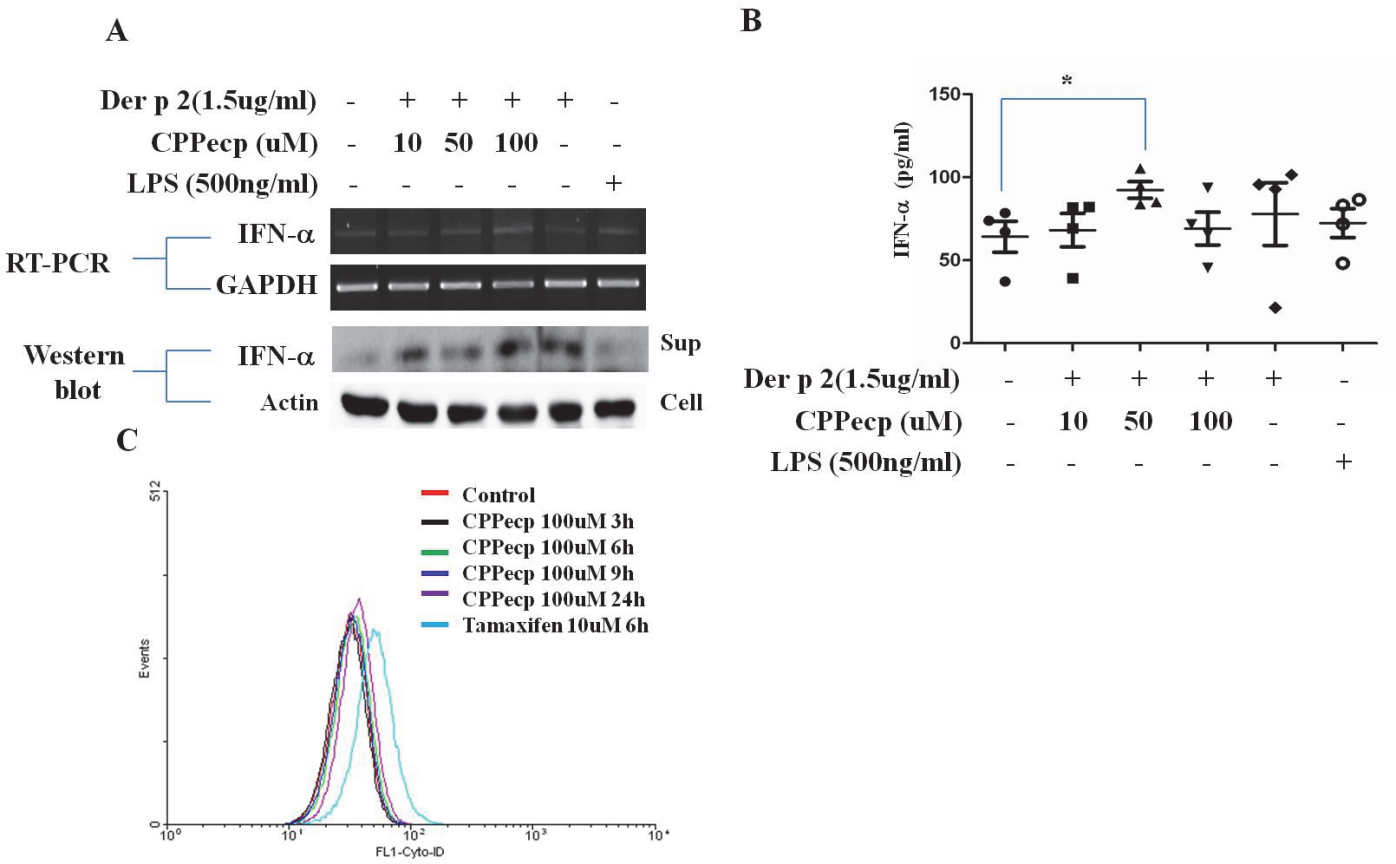
CPPecp upregulates IFN-β expression but not autophagy induction. (A) THP-1 cells were co-cultured with Der p 2 (1.5ug/ml) and CPPecp (10–100)uM for six hours, IFN-β expression was detect by RT-PCR and IFN-β secreted in the supernatant (sup) and β-actin in the cell lysates (cell) was detect by western blot. (B) CD14^+^ cells derived from HDM-allergic patients (n = 4) were co-cultured with Der p 2 (1.5ug/ml) and CPPecp 100uM for six hours. Culture supernatant were collected and expressions of IFN-β were detected by ELISA. Bars and error bars indicate mean and standard error of the mean (SEM), respectively.* p<0.05, compared to control. (C) THP-1 cells were treated with CPPecp 100uM over a time course. Autophagosomes were stained with Cyto-ID and fluorescence was detected by flowcytometry. Results shown are representative of three independent experiments.

It has been reported that inflammasome activation can be inhibited through upregulation of IFN-α production and autophagy. THP-1 cells were co-cultured with Der p 2 (1.5ug/ml) and different concentrations of CPPecp (10, 50, 100uM) for six hours and IFN-α expression was analyzed by RT-PCR and western blot. The results showed that IFN-α production was upregulated by co-cultured with 100uM CPPecp (Fig. 4A). However, THP-1 cells treated with different concentration of CPPecp (10, 50, 100 uM) alone would not increase IFN-a expression (S4 Fig.). We further analyzed level of IFN-β in the supernatant of CD14^+^ cells after stimulation. Levels of IFN-β were significantly upregulated in cells co-cultured with 50uM CPPecp and 1.5ug/ml of Der p 2 (Fig. 4B). The effect of CPPecp on autophagy induction on THP-1 cells was detected by flowcytometer and found Flowcytometer analysis showed that CPPecp had no effect on autophagy induction during the incubation period (Fig. 4C).

## Discussion

Innate immunity is the first line of defense against pathogens, and inflammasomes play an important role in innate immunity [26, 27]. It has been reported that viruses, air pollution or cigarette particles can activate inflammasomes [28–31]. To our knowledge, this is the first study that demonstrates a HDM major allergen can induce inflammasome activation. Inflammatory cells can release cytokines and chemokines to direct the migration of immune cells to infected or damaged tissues in order to eliminate foreign pathogens. Consequently, allergic asthma is caused by allergen-induced chronic inflammation. In this study, we demonstrated Der p 2 could upregulate proinflammatory cytokines in PBMCs derived from HDM allergic patients. These results suggest that proinflammatory cytokine production could be correlated with inflammasome activation. Inflammasomes are activated by two signals: 1) after the activation of toll-like receptors (TLR), gene transcriptions mediated by NF-kB increase and produce abundant pro-IL-1β. 2) After that, another signal from the activation of pro-caspase-1 originates from the inflammasome itself [32, 33]. In our previous study, we have demonstrated that Der p 2 can activate the signal transduction pathway of TLR-4 in B cells and also increase the expression of NF-kB [25]. In the present study, we demonstrated that the inflammasome activation in monocytes was upregulated after Der p 2 stimulation. Both the concentration and activity levels of caspase-1 were upregulated after Der p 2 stimulation. This finding was confirmed by a significant increase in IL-1β production. This also explains why allergens can induce chronic airway inflammation which can in turn lead to allergic asthma.

CPPs are designed to be able to mediate the conjugated cargo across the plasma membrane, making CPPs an effective and non-toxic mechanism for drug delivery [34]. However, few reports have investigated the real function of CPPs on target cells. Most studies focused on the efficiency of CPP transport of drugs into cells. In our previous reports, we showed that CPPecp has the ability to penetrate cells *in vivo* and *in vitro* [21]. Therefore, we decided to investigate the effects of CPPecp on immunomodulation. In this study, we demonstrated CPPecp can inhibit the inflammasone activation induced by Der p 2 and downregulate pro-inflammatory cytokine production. CPPecp inhibits inflammasome activation through upregulation of IFN-α production in monocytes. The mechanism of inhibition of inflammasome activation is through two pathways, one is by upregulation of IFN-α production and the other is by induction of autophagy [35, 36]. Type I INF signaling can directly inhibit NLRP3 inflammasomes activation in a STAT-1 dependent manner or induce IL-10 production which could activate STAT3 in an autocrine manner to reduce levels of pro-inflammatory cytokines [37]. On the other hand, autophagy is a cellular response to starvation as well as a quality-control system that can deliver damaged organelles and long-lived proteins from the cytoplasm to lysosomes for clearance [38, 39]. It has been reported that autophagy activation could limit the production of IL-1β by targeting ubiquitinated inflammasomes for destruction [35, 40]. However, in the present study CPPecp did not induce autophagy in monocytes during the incubation period. Thus, we believe the inhibitory effects of CPPecp on inflammasome activation were through upregulation of IFN-α production but not induction of autophagy.

In conclusion, our study demonstrated the mechanism of Der p 2 on inflammasome activation and the effects of CPPecp on immunomodulation. Thus, we suggest CPPecp can induce an anti- inflammatory immune response by inhibiting activation of inflammasomes; hence it has the potential to be a new anti-inflammatory agent for allergic asthma treatment in the future.

## Supporting Information

S1 Fig Effects of Der p 2 on pro-inflammatory cytokine expression in non- allergic patients. CD14^+^ cells derived from non-allergic patients (n = 2) were stimulated with Der p 2 (1.5ug/ml) for six hours; LPS (500ng/ml) was used as control. After stimulation, the culture supernatant was collected and protein levels of IL-1β, IL-6 and IL-8 were measured by ELISA. Bars and error bars indicate mean and standard error of the mean (SEM), respectively.
(TIF)

S2 Fig Effects of CPPecp on inflammasome activation and proinflammatory cytokine production. THP-1 cells were co-cultured with CPPecp (10, 50, 100 uM) for six hours; Der p 2 (1.5ug/ml) and LPS (500ng/ml) was used as control. Protein lysates were collected and expressions of NLRP3, ASC, caspase-1 were detected by Western blot (A). Culture supernatant was collected and the IL-1β and IL-6 concentration were measured by ELISA (B). Bars and error bars indicate mean and standard error of the mean (SEM), respectively. Results shown are representative of two independent experiments.
(TIF)

S3 Fig Effects of Der p 2 on pro-inflammatory cytokine expression in non- allergic patients. CD14^+^ cells derived from non-allergic patients (n = 2) were co-cultured with Der p 2 (1.5ug/ml) and CPPecp (10 to 100 uM) for 6 hours; mRNA expression of IL-1β, IL-6, IL-8 and GAPDH was detected by RT-PCR. Results shown are representative of two independent experiments.
(TIF)

S4 Fig Effects of CPPecp on IFN-α expression. THP-1 cells were cultured with CPPecp (10, 50, 100uM) for six hours. Der p 2 (1.5ug/ml) and and LPS (500ng/ml) was used as control. IFN-α expression was detected by RT-PCR and Western blot. Results shown are representative of two independent experiments.
(TIF)
