# Correction: Cell-Penetrating Peptide Derived from Human Eosinophil Cationic Protein Inhibits Mite Allergen Der p 2 Induced Inflammasome Activation

**DOI:** 10.1371/journal.pone.0127255

**Published:** 2015-04-24

**Authors:** Sheng-Jie Yu, En-Chih Liao, Meei-Ling Sheu, Dah-Tsyr Margaret Chang, Jaw-Ji Tsai

All instances of IFN-β in the published article should be replaced by IFN-α3.
