# Correction: Correction: Cell-Penetrating Peptide Derived from Human Eosinophil Cationic Protein Inhibits Mite Allergen Der p 2 Induced Inflammasome Activation

**DOI:** 10.1371/journal.pone.0129187

**Published:** 2015-06-01

**Authors:** Sheng-Jie Yu, En-Chih Liao, Meei-Ling Sheu, Dah-Tsyr Margaret Chang, Jaw-Ji Tsai

There is an error in the correction published on April 24, 2015. All instances of IFN-β in the published article should be replaced by IFN-α.
