# Supplementary figures and images for: Cell-Penetrating Peptide Derived from Human Eosinophil Cationic Protein Inhibits Mite Allergen Der p 2 Induced Inflammasome Activation
Source: PLoS One. 2015 Mar 25;10(3):e0121393. doi: 10.1371/journal.pone.0121393 (PMC4373874; doi:10.1371/journal.pone.0121393)

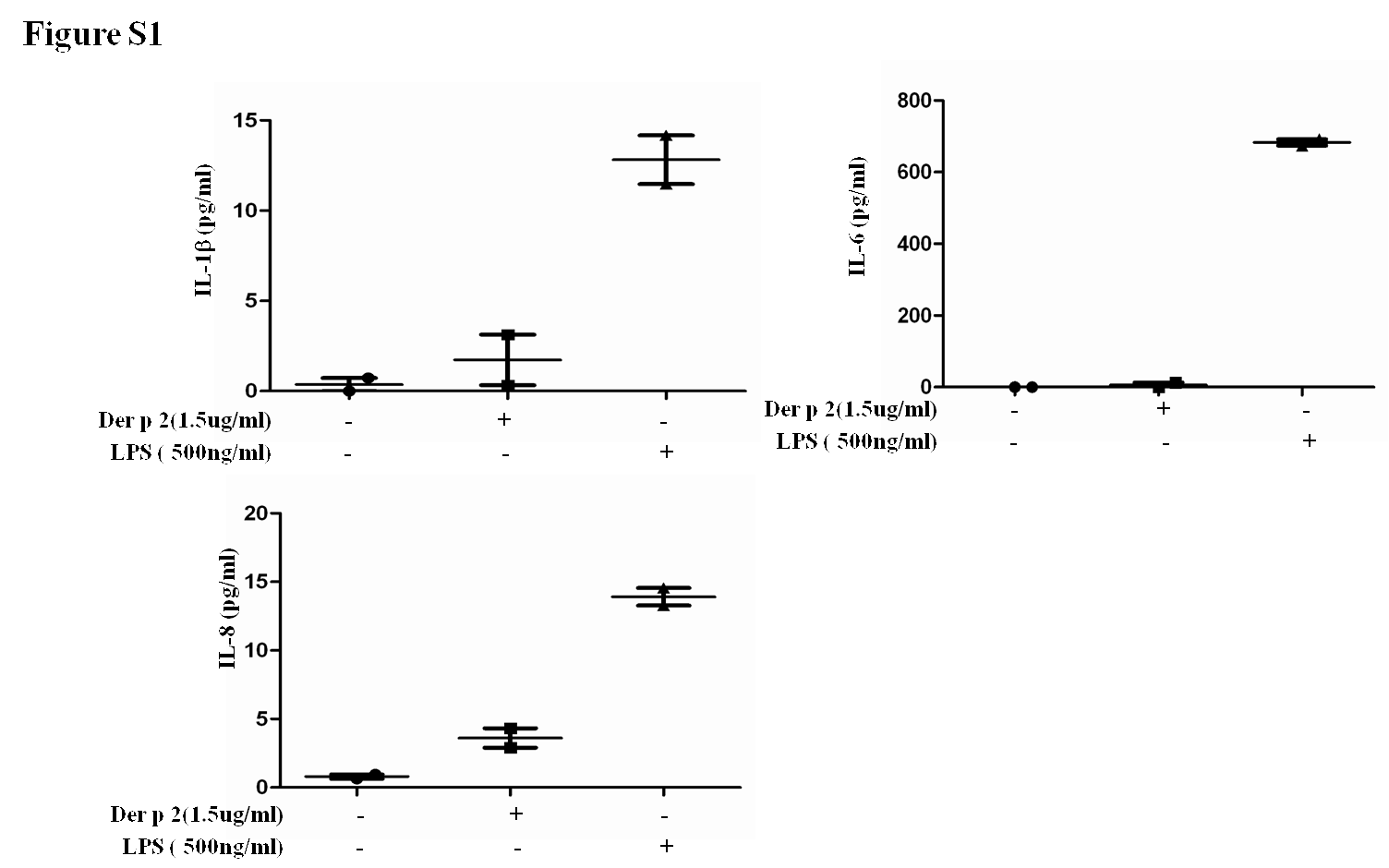

Supplement: S1 Fig — CD14+ cells derived from non-allergic patients (n = 2) were stimulated with Der p 2 (1.5ug/ml) for six hours; LPS (500ng/ml) was used as control. After stimulation, the culture supernatant was collected and protein levels of IL-1β, IL-6 and IL-8 were measured by ELISA. Bars and error bars indicate mean and standard error of the mean (SEM), respectively. (TIF) [file pone.0121393.s001.tif]

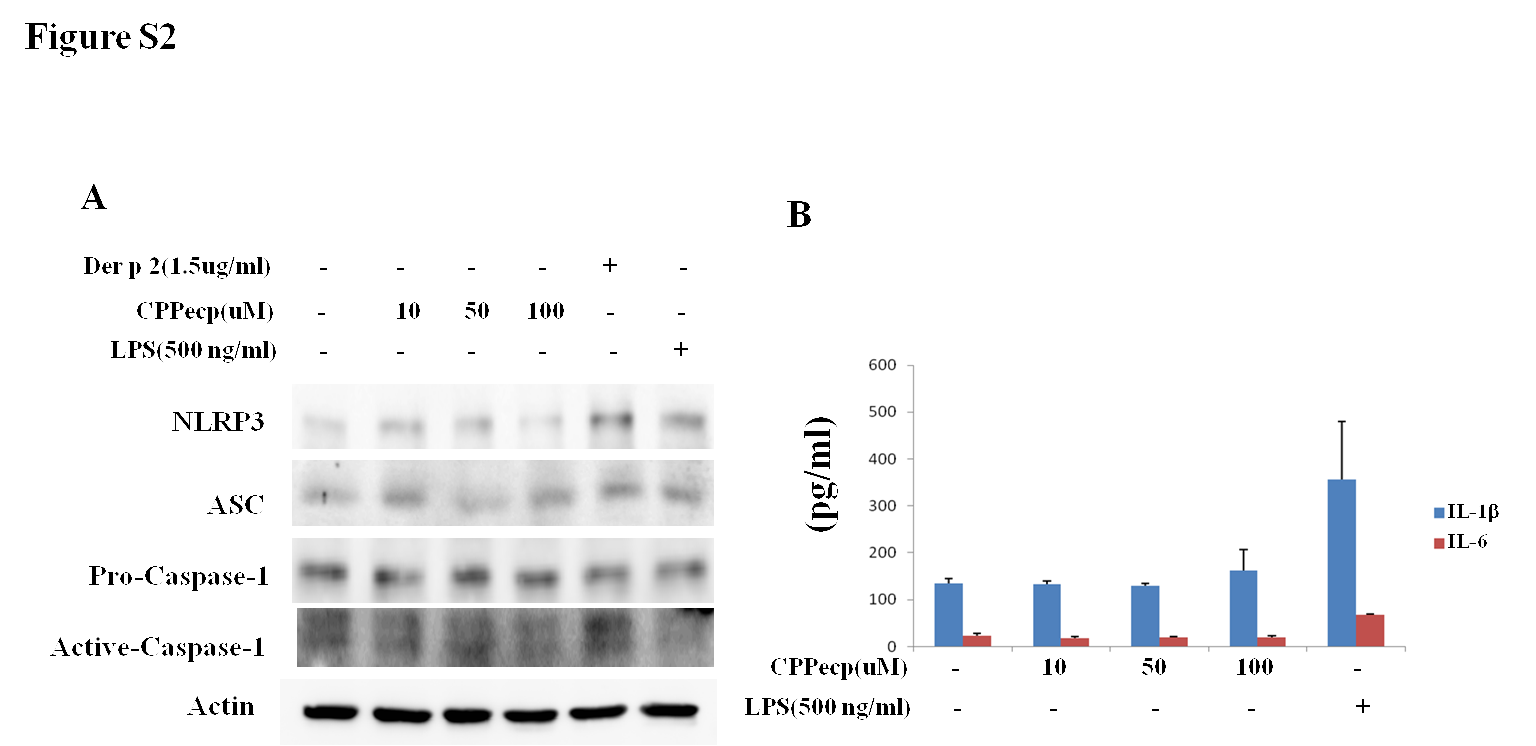

Supplement: S2 Fig — THP-1 cells were co-cultured with CPPecp (10, 50, 100 uM) for six hours; Der p 2 (1.5ug/ml) and LPS (500ng/ml) was used as control. Protein lysates were collected and expressions of NLRP3, ASC, caspase-1 were detected by Western blot (A). Culture supernatant was collected and the IL-1β and IL-6 concentration were measured by ELISA (B). Bars and error bars indicate mean and standard error of the mean (SEM), respectively. Results shown are representative of two independent experiments. (TIF) [file pone.0121393.s002.tif]

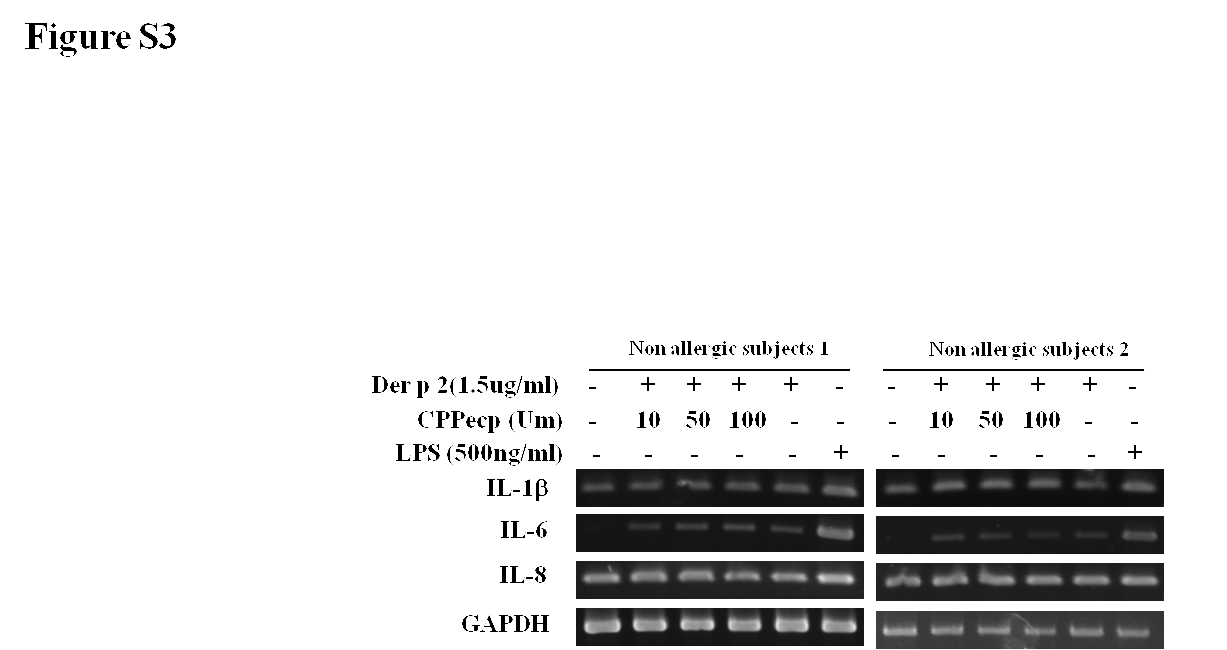

Supplement: S3 Fig — CD14+ cells derived from non-allergic patients (n = 2) were co-cultured with Der p 2 (1.5ug/ml) and CPPecp (10 to 100 uM) for 6 hours; mRNA expression of IL-1β, IL-6, IL-8 and GAPDH was detected by RT-PCR. Results shown are representative of two independent experiments. (TIF) [file pone.0121393.s003.tif]

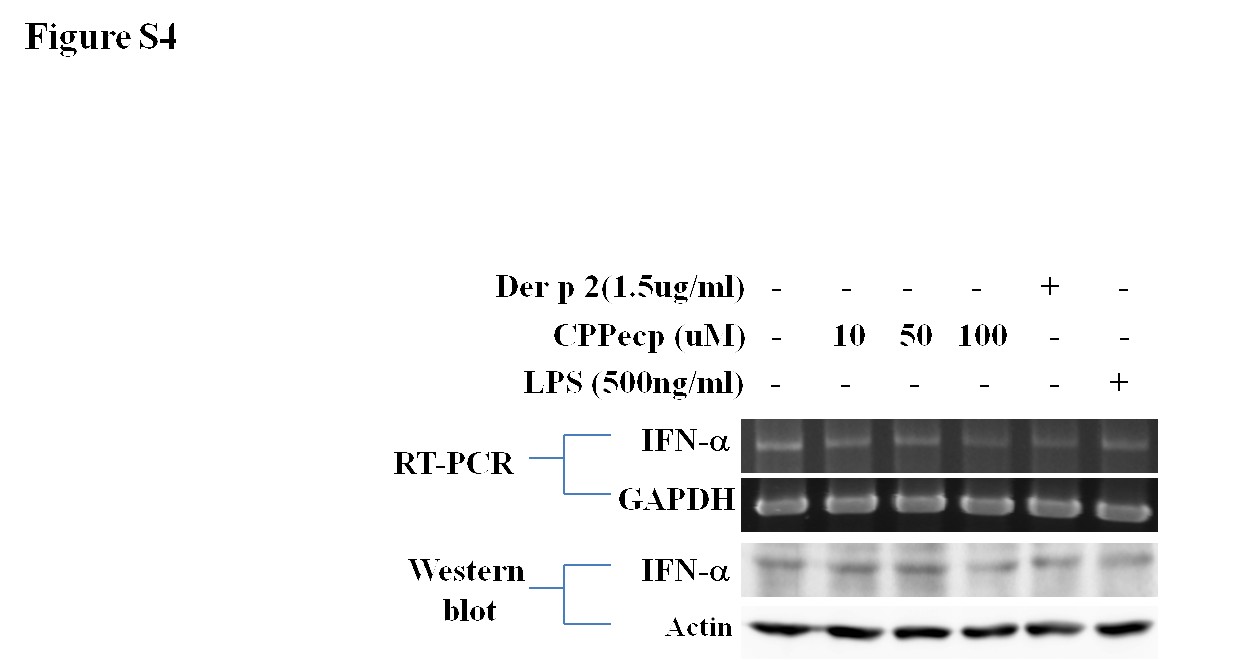

Supplement: S4 Fig — THP-1 cells were cultured with CPPecp (10, 50, 100uM) for six hours. Der p 2 (1.5ug/ml) and and LPS (500ng/ml) was used as control. IFN-α expression was detected by RT-PCR and Western blot. Results shown are representative of two independent experiments. (TIF) [file pone.0121393.s004.tif]
